# Mechanism underlying the immune checkpoint inhibitor-induced hyper-progressive state of cancer

**DOI:** 10.20517/cdr.2021.104

**Published:** 2022-02-08

**Authors:** Peng Ding, Lu Wen, Fan Tong, Ruiguang Zhang, Yu Huang, Xiaorong Dong

**Affiliations:** Cancer Center, Union Hospital, Tongji Medical College, Huazhong University of Science and Technology, Wuhan 430022, Hubei, China.; ^#^Authors contributed equally.

**Keywords:** Immune checkpoint inhibitors, hyper-progressive disease, immunotherapy, tumor microenvironment

## Abstract

Immune checkpoint inhibitors (ICIs) are gradually replacing chemotherapy as the cornerstone of the treatment of advanced malignant tumors because of their long-lasting and significant effect in different tumor types and greatly prolonging the survival time of patients. However, not all patients can respond to ICIs, and even rapid tumor growth after treatment with ICI has been observed in a number of clinical studies. This rapid progression phenomenon is called hyper-progressive disease (HPD). The occurrence of HPD is not uncommon. Past statistics show that the incidence of HPD is 4%-29% in different tumor types, and the progression-free survival and overall survival of patients with HPD are significantly shorter than those of the non-HPD progressor group. With the deepening of the study of HPD, we have established a preliminary understanding of HPD, but the diagnostic criteria of HPD are still not unified, and the addition of biomarkers may break this dilemma. In addition, quite a few immune cells have been found to be involved in the occurrence and development of HPD in the tumor microenvironment, indicating that the molecular mechanism of HPD may be triggered by a variety of ongoing events at the same time. In this review, we summarize past findings, including case reports, clinical trials, and fundamental research; compare the diagnostic criteria, incidence, and clinical prognostic indicators of HPD in different studies; and explore the molecular mechanism and future research direction of HPD.

## INTRODUCTION

Immune checkpoint inhibitors (ICIs) have continuously promoted the progress of the treatment of malignant tumors since their advent. They have gradually replaced chemotherapy as the cornerstone for the treatment of malignant tumors; however, ICIs are only effective in some patients and remain ineffective in most populations. Changes in the tumor microenvironment (TME) induced by ICIs stimulate the accelerated growth of malignant tumor cells. This special tumor progression mode is called the hyper-progressive disease (HPD) state. Lahmar *et al*.^[1]^ reported the HPD phenomenon for the first time in a wall newspaper at the 2016 European Society of Medical Oncology Annual Meeting. Eight patients with advanced non-small cell carcinoma (NSCLC) exhibiting fast progression at the time of initial examination were identified as HPD cases. HPD gained attention in 2017 when Champiat *et al*.^[2]^ reported a 9% HPD incidence in 131 cancer patients in a phase I prospective study. Evidence of HPD, the phenomenon of early crossover of the survival curve, is also reported in some phase III clinical studies, including in NSCLC (CheckMate026^[3]^, CheckMate057^[4]^, and CheckMate227^[5]^), HNSCC (CheckMate141^[6]^), and uroepithelial carcinoma (Keynote045^[7]^ and IMvigor211^[8]^). Patients receiving immunotherapy died at a greater rate in the first three months than those treated with chemotherapy. HPD is not unique to immunotherapy and can also be caused by chemotherapy^[9]^ and targeted therapy^[10]^. However, the incidence of HPD after ICI treatment is significantly higher than in the chemotherapeutic regime^[11]^. Since its discovery in 2016, several studies on HPD have been reported in the last five years. Nevertheless, the incidence, diagnostic criteria, and pathogenesis of HPD remain in the preliminary stages. This review summarizes the recently published cases, clinical studies, and basic studies on HPD.

## DIAGNOSTIC CRITERIA FOR HPD

At present, there is no agreement on the diagnostic criteria of HPD. Although many clinical studies on HPD adopt different diagnostic criteria, the diagnostic indicators of HPD mainly focus on the following five: tumor growth rate (TGR), ΔTGR, tumor growth kinetics (TGK), Response Evaluation Criteria in Solid Tumors (RECIST), and time to failure (TTF). TGR represents the percentage of monthly tumor volume growth (excluding new and immeasurable lesions), and the difference between the two at and before treatment is defined as ΔTGR. TGK is defined similarly to TGR, but it primarily reflects tumor growth rate per unit time. TTF refers to the time of treatment failure. Champiat *et al*.^[2]^ earlier adopted such criteria as TGR > 2 and RECIST to assess the progress for the first time to define HPD. In the same year, Ferrara *et al*.^[9]^ used a different cut-off value of ΔTGR > 50%. Kato *et al*.^[12]^ added TTF < 2 months on the basis of predecessors. Saâda-Bouzid *et al*.^[13]^ used a new index, TGK, as a measure of tumor growth rate that may be more appropriate to define HPD. Kim *et al*.^[14]^ reviewed the survival time of 335 patients with advanced NSCLC who received ICI monotherapy; it was proved that HPD defined by volume measurement (TTF < 2 months, TGK > 2, and ΔTGR > 50%) is more accurate than that defined based on one-dimensional analysis (RECIST 1.1). Kas *et al*.^[15]^ conducted a retrospective study of 406 patients with advanced NSCLC treated with ICIs. They calculated their results using the different definitions of five clinical studies. The incidence of HPD ranged from 5.4% to 18.5%, and the median survival ranged from 3.4 to 6.0 months. ΔTGR was found to be most correlated with poor prognosis, and ΔTGR > 100% was updated as the optimal threshold.

Although the volumetric method is superior to the RECIST standard, there are practical problems: first, not all patients can complete the pre-baseline computed tomography (CT) scan, especially those receiving ICI as late first-line treatment. Second, new and unmeasurable lesions cannot be measured by TGR. Matos *et al*.^[16,17]^ returned to RECIST standard and proposed a new method to define HPD: (1) target lesions increased by more than 40% from baseline; and/or (2) target lesions increased by more than 20% from baseline and new lesions appeared in at least two different organs. The overall survival (OS) of the HPD group using the new standard decreased significantly, which was statistically significant, compared to the non-HPD group, whereas the OS of the HPD group using TGR decreased, but not statistically significantly. However, Gomes da Morais *et al*.^[18]^ reviewed the literature and compared the main criteria of HPD proposed by Ferté, Le Tourneau, Garralda, and Caramella. These criteria include ΔTGR > 100 (Caramella) and 20% target lesion progression plus the occurrence of new lesions in at least two different organs. The incidence of HPD was 23.9%, 23.9%, 32.4% and 8.4%, respectively. They believed that the Caramella standard has low sensitivity; the Garralda standard has low specificity; and the Le Tourneau and Ferté standards seem to have similar performance in detecting HPD, but, from a practical point of view, the two-dimensional evaluation of TGK (Le Tourneau) is easier than the three-dimensional evaluation of TGR (Ferté). The importance of pre-baseline CT scanning in diagnosing HPD was thus highlighted, but only 71 eligible patients were enrolled in this study. Later, Abbar *et al*.^[19]^ expanded the study to 169 advanced NSCLC patients treated with ICI; the incidence of HPD (11.3%, 5.7%, 17.0%, 9.6% and 31.7%) was calculated based on five indicators. In addition to the discovery of large heterogeneity, the definition of HPD based on TTF standard was correlated with OS, while the other diagnostic criteria were not correlated with OS.

Thus, combining indicators with each other may be more conducive to diagnosis. The radiological and clinical diagnostic criteria for HPD are still being explored. With the deepening of the understanding of biomarkers for HPD, biomarkers may be involved in the diagnostic criteria of HPD in the future, and the joint definition of HPD by three diagnostic methods may be more accurate and practical.

## INCIDENCE AND PROGNOSTIC INDICATORS OF HPD

The incidence and clinical prognostic indicators of HPD are also different. Chen *et al*.^[20]^ reviewed the medical records of 377 patients with multiple malignancies and reported the incidence of HPD (10.08%). Factors associated with HPD include the presence of more than two metastatic sites, Eastern Cooperative Oncology Group score ≥ 2, liver metastasis, and lactic dehydrogenase level higher than the normal upper limit. Kirsten rat sarcoma viral oncogene homolog status is significantly correlated with HPD in colon cancer patients. Two large-scale meta-analyses reported the incidence of HPD in patients with pan-cancer as 1%-30%^[21]^ and 5.9%-43.1%^[22]^. The clinical prognostic markers used in these analyses were similar to those reported by Chen *et al*.^[20]^. Ferrara *et al*.^[9]^, using RECIST 1.1 and TGR criteria, reported a 13.8% (56/406) HPD incidence in patients with advanced NSCLC; HPD was associated with more than two metastases before immunotherapy. Kim *et al*.^[23]^ first defined three criteria (TGR, TGK, and TTF) to calculate the incidence of HPD (20.9%, 20.5%, and 37.3%, respectively). In HPD patients who satisfied both TGR and TGK criteria, poorer progression-free survival (PFS) and OS were observed. Although no clinicopathological variables of HPD were reported in the study, in the exploratory biomarker analysis of peripheral blood, CD8^+^ T lymphocytes, lower effector/memory subsets (CCR7^-^CD45RA^-^ T cells in total CD8^+^ T cells), and higher populations of severely depleted cells (TIGIT^+^ T cells in PD-1^+^CD8^+^ T cells) were associated with HPD and poor survival. In two real-world studies, the incidence of HPD in advanced NSCLC was 19.2% (16/83)^[24]^ and 8.1% (6/74)^[25]^. Among them, one study reported an increased rate of fluid accumulation (up to 90%) and decreased albumin level, while the other showed a significant increase in the number of circulating Treg cells in HPD patients. Chen *et al*.^[26]^ performed a meta-analysis consisting of 1389 NSCLC patients from six clinical studies and found that the incidence of HPD was 8.02%-30.43%. The incidence of HPD and clinical prognostic indicators in cancer types are shown in Table 1.

**Table 1:** Recent retrospective studies on hyper-progression after immunotherapy

**Tumor type**	**Agents**	**HPD criteria**	**HPD incidence**	**Prognostic indicators**	**Outcomes (HPD *vs.* non-HPD)**	**Ref.**
Multiple tumor types	PD-1/PD-L1 inhibitor monotherapy	-	1%-30% (217/1519)	Serum LDH > upper normal limit; > 2 metastatic sites prior to immunotherapy; liver metastatic sites; RMH prognostic score ≥ 2; positive PD-L1 expression status	-	Kim *et al*.^[21] ^(2019)
Multiple tumor types	PD-1/PD-L1 inhibitor monotherapy	RECIST criteria (1.4× baseline sum target lesions or 1.2× baseline sum target lesions + new lesions in at least 2 different organs) or TGR ≥ 2	RECIST criteria, 10.7% (29/270); TGR criteria, 6.3% (14/221)	RECIST criteria of no or TGR criteria of liver metastatic sites; > 2 metastatic sites prior to immunotherapy	OS: 5.23 months *vs.* 7.33 months, *P = *0.04, by RECIST; 4.2 months *vs.* 6.27 months, *P = *0.346, by TGR	Matos *et al*.^[17]^ (2020)
Multiple tumor types	PD-1 inhibitors (nivolumab or pembrolizumab)	ΔTGR > 50%	10.08% (38/377)	> 2 metastatic sites prior to immunotherapy; ECOG ≥ 2; hepatic metastases; serum LDH > upper normal limit; KRAS status in colorectal cancer	OS: 3.6 months *vs.* 7.3 months, *P < *0.01	Chen *et al*.^[20] ^(2021)
Multiple tumor types	PD-1 or PD-L1 inhibitor monotherapy or combined with CTLA-4 inhibitor	4 categories (TGR, TGK, early tumor burden increase, or combinations of the above)	5.9%-43.1% (3109)	-	-	Park *et al*.^[22] ^(2021)
NSCLC	PD-1 or PD-L1 inhibitor monotherapy or combined with CTLA-4 inhibitor	RECIST 1.1 progression and ΔTGR > 50%	14% (56/406 treated with ICI); 5% (3/59 treated with chemotherapy)	> 2 metastatic sites prior to immunotherapy	OS: HR = 2.18, 95%CI: 1.29-3.69, *P = *0.03	Ferrara *et al*.^[9] ^(2018)
NSCLC	PD-1 inhibitors (nivolumab)	< 3 nivolumab injections	20% (57/292)	PS > 2 at nivolumab initiation	OS: 1.4 months *vs.* 13.5 months, *P* < 0.0001	Costantini *et al*.^[112] ^(2019)
NSCLC	PD-1 or PD-L1 inhibitor monotherapy	Volumetric time-dependent criteria (TGK ≥ 2) or one-dimensional criteria: RECIST 1.1 progression	14.3% (48/335 by volumetric assessment); 13.1% (44/335 by one-dimensional criteria)	High neutrophil-to-lymphocyte ratio; LKB1 mutation	OS: 4.7 months *vs.* 7.9 months, *P* = 0.009, by volumetric; 5.2 months *vs.* 7.1 months, *P* = 0.288, by RECIST	Kim *et al*.^[14] ^(2020)
NSCLC	PD-1 or PD-L1 inhibitor monotherapy	TGK ≥ 2, TGR ≥ 2, or TTF < 2 months	20.9% (55/263 TGK), 20.5% (54/263 TGR), 37.3% (98/263 TTF)	≥ 2 metastatic locations; liver metastases; neutrophils; neutrophil-to-lymphocyte ratio; LDH; high CD8^+^PD-1^+^TIGIT^+^ T cells; low CD8^+^CCR7^-^CD45RA^-^ T cells	PFS: HR = 4.62, 95%CI: 2.87-7.44, *P < *0.05; OS: HR = 5.71, 95%CI: 3.14-8.23, *P* < 0.05	Kim *et al*.^[23] ^(2019)
NSCLC	PD-1 inhibitors (nivolumab)	RECIST 1.1 progression and TGR ≥ 2	19.2% (16/83)	Pleura or pericardium metastasis; low circulating albumin	PFS: 0.43 months *vs.* 1.35 months; OS: 2.2 months *vs.* 4.1 months	Kim *et al*.^[24]^ (2020)
NSCLC	PD-1 /PD-L1 inhibitor monotherapy or combined with other immunotherapy treatments	Ferté criteria (RECIST 1.1 progression and TGR ≥ 2), Le Tourneau criteria (TGK > 2), Garralda criteria (increase of ≥ 20% in target tumor burden plus multiple new lesions or increase of ≥ 40% in target tumor burden compared with baseline) or Caramella criteria (RECIST 1.1 progression and ΔTGR > 100%)	5.4%-18.5% (406)	No (including previously described prognostic factors such as age, LDH, albumin, > 2 metastatic sites, RMH score)	-	Kas *et al*.^[15] ^(2020)
NSCLC	PD-1 /PD-L1 inhibitor monotherapy or combined with other immunotherapy treatments	-	8.02%-30.43% (1389)	ECOG > 1; RMH ≥ 2; serum LDH > upper Normal limit; > 2 metastatic sites prior to immunotherapy; liver metastases	-	Chen *et al*.^[26] ^(2020)
NSCLC	PD-1 or PD-L1 inhibitor monotherapy or combined with CTLA-4 inhibitor	5 definitions (TGR, ΔTGR, TGK, RECIST, or TTF)	11.3%, 5.7%, 17%, 9.6%, 31.7% (169)	-	-	Abbar *et al*.^[19] ^(2021)
NSCLC	PD-1 or PD-L1 inhibitor monotherapy	TGK > 2 and TTF ≤ 2 months	11.3% (26/231)	Heavy smoker; PD-L1 expression ≤ 1%; ≥ 3 metastatic sites	OS: 5.5 months *vs.* 6.1 months	Kim *et al*.^[110] ^(2021)
NSCLC	PD-1/PD-L1 inhibitor monotherapy or combined with chemotherapy	TGR > 2	17.6% (25/142 monotherapy); 2.9% (1/34 combination therapy)	-	-	Matsuo *et al*.^[113]^ (2021)
NSCLC	PD-1 or PD-L1 inhibitor monotherapy	TGK ≥ 2	8.1% (6/74)	CD4^+^CD25^+^CD127^lo^FoxP3^+^ Treg cells was increased on Day 7 after initiation of treatment	-	Kang *et al*.^[25] ^(2021)
HNSCC	PD-1 or PD-L1 inhibitor monotherapy or combined with CTLA-4 inhibitor	TGK > 2	14.4% (18/125)	Younger age; primary tumor of oral cavity; previous locoregional irradiation	PFS: 1.2 months *vs.* 3.4 months, *P* < 0.001; OS: 3.4 months *vs*. 10.7 months, *P* = 0.047	Park *et al*.^[31] ^(2020)
HNSCC	PD-1 or PD-L1 inhibitor monotherapy or combined with CTLA-4 inhibitor	TGK ≥ 2	15.4% (18/117)	Primary site in the oral cavity; administration of ICI in the second/third setting	PFS: 1.8 months *vs.* 6.1 months, *P* = 0.0001; OS: 6.53 months *vs.* 15 months, *P* = 0.0018	Economopoulou *et al*.^[51] ^(2021)
MM	PD-1 inhibitor, CTLA-4 inhibitor monotherapy or combination	TTF < 2 months, doubling of tumor burden, and TGR > 2	1.3% (1/75)	-	-	Schuiveling *et al*.^[114] ^(2021)
GC	PD-1 inhibitors (nivolumab)	TGK ≥ 2 and (S_POST_/S_0_-1) > 0.5	22.1% (143)	PD-L1 CPS; MMR	PFS: 1.2 months *vs.* 1.7 months, *P* < 0.001; OS: 3.3 months *vs.* 6.8 months, *P* = 0.012	Hagi *et al*.^[115]^ (2020)
HCC	PD-1 inhibitors (nivolumab)	TGK > 4 and ΔTGR > 40%	12.7% (24/189)	Neutrophil-to-lymphocyte ratio	PFS: HR = 2.194, 95%CI: 1.214-3.964; OS: HR = 2.238, 95%CI: 1.233-4.062	Kim *et al*.^[116] ^(2021)
RCC and UC	PD-1/PD-L1 inhibitor monotherapy	Tumor burden increase ≥ 50%, TGR ≥ 2, or ≥ 10 metastatic sites	0.9% (1/102), 11.9% (12/101)	UC; creatinine > 1.2 mg/dL	PFS: 1.3 months *vs.* 3.9 months, *P* < 0.001; OS: 3.5 months *vs.* 7.3 months, *P* < 0.001	Hwang *et al*.^[117] ^(2020)
GYN	PD-1 inhibitor	Tumor burden increase of ≥ 40% or tumor burden increase of ≥ 20% plus multiple new lesions	23.3% (14/60)	Neutrophil-to-lymphocyte ratio; > 3 metastatic sites	-	Rodriguez Freixinos *et al*.^[118]^ (2018)

PFS: Progression-free survival; OS: overall survival; NSCLC: non-small-cell carcinoma; HNSCC: head and neck squamous cell carcinoma; MM: malignant melanoma; GC: gastric cancer; HCC: hepatocellular carcinoma; RCC: renal cell carcinoma; UC: urothelial carcinoma; GYN: gynecological malignancies; PD-1/PD-L1: programmed cell death-1/programmed cell death-ligand 1; LDH: lactic dehydrogenase; RMH: Royal Marsden Hospital score; RECIST: Response Evaluation Criteria in Solid Tumors; TGR: tumor growth rate; ΔTGR: the difference of TGR before and during immunotherapy; TGK: tumor growth kinetics; TTF: time to treatment failure; ECOG: Eastern Cooperative Oncology Group; KRAS: Kirsten rat sarcoma viral oncogene homolog; CPS: combined positive score of PD-L1 expression; MMR: mismatch repair.

## CASE SUMMARY

The limitations of ICIs, as they may not be appropriate for some patients, caused “disease flare” in a 54-year-old man with stage IIB lung adenocarcinoma after 10th-line treatment with nivolumab^[27]^. This case opened up the HPD patient reports, and, according to incomplete statistical data, in 44 cases involving 53 patients, malignant tumor types were mainly distributed in the respiratory system, digestive system, and urinary system and were immune to single and double drugs to a significantly higher degree than due to the immune or anti-angiogenesis drugs with combination chemotherapy. Most patients with HPD after ICI treatment developed liver, lung, and brain metastases. Selected case studies are listed in Table 2. Among them, the youngest patient was a 13-year-old girl suffering from malignant melanoma, which progressed to HPD mode after two cycles of treatment with avelumab in palliative radiotherapy. The Food and Drug Administration has approved ICIs for the treatment of children with microsatellite unstable malignant tumors based on reports in adults^[28]^. However, the interaction between children’s immune systems and anti-PD1 therapy remains unclear. The oldest patient was an 80-year-old patient with lung squamous carcinoma^[29]^. The symptoms of HPD were pneumonia, pleural effusion, and pericardial effusion. Many patients developed the same symptoms after ICI treatment for malignant tumors of the respiratory system and digestive system and malignant melanoma. A previous study in South Korea reported a higher frequency of increased fluid accumulation in HPD patients with pleural or pericardial metastases after treatment with nivolumab as compared to the progressive disease (PD) patients without HPD [90% (9/10) *vs.* 28.6% (4/14); *P* = 0.005]; the circulating albumin level was significantly reduced in HPD patients (*P* = 0.030)^[24]^. A considerable proportion of HPD occurred in patients after radiotherapy, which suggested that radiotherapy had a bidirectional regulatory effect on the anti-tumor immune response. If the immunosuppressive function of radiotherapy is dominant, a combination of ICIs may lead to HPD^[30]^. A clinical study of head and neck squamous cell carcinoma also suggested that previous local irradiation was an important predictor of HPD^[31]^. In addition to being associated with radiotherapy, AKT1 E17K mutation^[32]^ and PI3K/AKT pathway^[33]^ were also related to HPD. Interestingly, after immunohistochemical staining of the primary tumor and metastases samples with HPD, Barham *et al*.^[34]^ showed that the tumor infiltrating lymphocyte (TIL) number was not necessarily correlated with ICI response, as levels of granzyme B and TIA-1 of infiltrated CD8^+^ T cells were mostly negative, indicating that these were inflammatory T cells which cause tumor drug resistance and myocarditis. They cannot effectively dissolve the tumor, so additional functional markers are required to distinguish between inflammatory and cytolytic CD8^+^ TIL. For treatment, the salvage therapy in HPD has not been limited to chemotherapy. A patient with lung adenocarcinoma developed HPD with rib metastasis shortly after ICI-based combination therapy, and the lesion was significantly reduced after implantation of I^125^ particles into the chest wall^[35]^. Another patient with lung adenocarcinoma showed MET amplification on re-biopsy after HPD and remission occurred with a c-MET inhibitor^[36]^. A patient with triple-negative breast cancer showed HPD after pembrolizumab treatment combined with chemotherapy and remission with atezolizumab administration combined with chemotherapy^[37]^. A patient with cardiac cancer was in remission after salvage therapy with paclitaxel and ramucirumab following HPD^[38]^.

**Table 2:** Cases summary on hyper-progression after immunotherapy

**Tumor type**	**Gender**	**Age (years)**	**Agents**	**Radiotherapy before ICIs**	**Clinical symptoms**	**Progressive organ**	**Ref.**
SCLC	Male	35	Nivolumab	No	Pleural effusion	Chest wall	Chiba *et al*.^[119] ^(2020)
LUSC	Male, Male	69, 80	Nivolumab	No	Pneumonia, pleural effusion, pericardial effusion	Lung	Kanazu *et al*.^[29] ^(2018)
LUAD	Female	66	Pembrolizumab	Yes	Pleural effusion, pericardial effusion	Brain, lung	Fricke *et al*.^[120] ^(2020)
LUAD	Male	68	Nivolumab	No	Jaundice, fever	Liver, pancreas	Martorana *et al*.^[121] ^(2021)
LUAD	Female	63	Sintilimab	Yes	Abdominal distension, poor appetite	Liver, pancreas	Lin *et al*.^[122] ^(2020)
LUAD	Male	65	Pembrolizumab and paclitaxel liposome (salvage treatment: c-Met inhibitor)	Yes	-	Brain, lung	Peng *et al*.^[36] ^(2020)
LPC	Male	66	Atezolizumab	Yes	Pericardial effusion, pericarditis, pleural effusion	Lung, brain, liver, diaphragm	Oguri *et al*.^[123] ^(2021)
ESCC	Male	40	Camrelizumab	No	-	Liver	Wang *et al*.^[124] ^(2020)
GC	Male	36	Nivolumab (salvage treatment: capecitabine and pyrotinib)	No	-	Lung, liver	Huang *et al*.^[125] ^(2019)
AEG	Female	56	Pembrolizumab (salvage treatment: paclitaxel and ramucirumab)	No	-	Lung, spine, ilium, retroperitoneal lymph node, *etc*.	Sama *et al*.^[38] ^(2019)
HCC	Male	36	Atezolizumab and bevacizumab	No	Abdominal pain	Liver	Singh *et al*.^[126] ^(2021)
HCC	Male/Male/Male	69/72/69	Tremelimumab/nivolumab/tremelimumab and durvalumab	No/TARE/TARE	-	Liver, portal vein thrombosis/lung, peritoneum/liver, lung	Wong *et al*.^[127] ^(2019)
COAD	Female	48	Pembrolizumab	No	Fatigue	Liver, retroperitoneal lymph node	Chan *et al*.^[128] ^(2020)
CMM	Female	25	Nivolumab	Yes	Ascites, pleural effusion, epilepsy	Peritoneum, pleura, brain	Yilmaz *et al*.^[129] ^(2019)
AMM	Female	49	Ipilimumab and nivolumab (salvage treatment: chemotherapy)?	No	-	Lung, brain	Forschner *et al*.^[130] ^(2017)
MMM	Female	79	Ipilimumab and nivolumab	Yes	Fulminant myocarditis, ascites, dizzy	Lung, peritoneum	Barham *et al*.^[34] ^(2021)
MM	Female	13	Nivolumab	Yes	-	Multiple organs	Vaca *et al*.^[28] ^(2019)
IBC	Male	78	Nivolumab	Yes	-	Sternum, liver	Koukourakis *et al*.^[131] ^(2020)
KIRC	Female	42	Nivolumab	Yes	Arthritis of hand and knee	Lung	Liu *et al*.^[30] ^(2021)
mUC	Male	57	Anti-PD-L1 and immune checkpoint modulator	No	-	Liver, brain	Grecea *et al*.^[132] ^(2020)
CSEC	Female	46	Pembrolizumab	Yes	Biliary obstruction	Liver	Lin *et al*.^[122] ^(2020)
SCCC	Female	49	Pembrolizumab	No	-	Lung	Xu *et al*.^[32] ^(2019)
PM	Male	75	Nivolumab	No	Abdominal distension	Liver	Ikushima *et al*.^[133] ^(2020)
TNBC	Female	67	Pembrolizumab and gemcitabine (salvage treatment: atezolizumab and nab-paclitaxel)	No	Fatigue, poor appetite, abdominal pain	Liver	Feng *et al*.^[37] ^(2021)
MSC	Female	60	Nivolumab	No	Decreased eyesight	Orbit, brain	Xiang *et al*.^[134] ^(2020)
LS	Male	63	Durvalumab and tremelimumab	Yes	-	Liver	Chan *et al*.^[135] ^(2020)

SCLC: Small cell lung cancer; LUSC: lung squamous cell carcinoma; LUAD: lung adenocarcinoma; LPC: lung pleomorphic carcinoma; ESCC: esophageal squamous cell carcinoma; GC: gastric cancer; AEG: adenocarcinoma of esophagogastric junction; HCC: hepatocellular carcinoma; COAD: colon adenocarcinoma; CMM: cutaneous malignant melanoma; AMM: acral malignant melanoma; MMM: mucosal malignant melanoma; MM: malignant melanoma; IBC: invasive bladder cancer; KIRC: kidney renal clear cell carcinoma; mUC: metastatic urothelial cancer; CSEC: cervical squamous epithelium carcinoma; SCCC: small cell carcinoma of cervix; PM: peritoneal mesothelioma; TNBC: triple-negative breast cancer; MSC: maxillary sinus carcinoma; LS: liposarcoma.

## MOLECULAR MECHANISM UNDERLYING HPD

The mechanism of action underlying ICI is the removal of the “braking” function of immune checkpoints and reduction in the escape of tumor cells to enhance the anti-tumor immune response of effector T cells^[39]^. ICIs reverse the immunosuppressive state of T cells by disrupting the programmed cell death-1/programmed cell death-ligand 1 (PD-1/PD-L1) axis^[40]^. However, PD-1 receptors are present not only on the surface of T cells but also on the surface of many innate or acquired immune cells, including NK cells, monocytes, macrophages, Treg cells, and B cells^[41]^. Furthermore, immune cells have varying impacts on PD-1/PD-L1 axis disruption, boosting or inhibiting immune function. In addition, tumor treatment through ICI intervention may also induce changes in the oncogenic pathways of the tumor cells and result in their rapid proliferation and spread^[42]^. Therefore, HPD may not be triggered by a single factor, but by a series of events that occur simultaneously. Most of the current studies on the molecular mechanisms of HPD focus on the tumor and the tumor microenvironment. In the next sections, we discuss these in detail to facilitate the understanding of the molecular mechanisms underlying ICI-induced HPD. The molecular mechanisms underlying HPD are shown in Table 3.

**Table 3:** Mechanisms summary on hyper-progression after immunotherapy

**Tumor cells**	**Tumor microenvironment**	
1. Loss of expression of tumor-associated antigens^[43]^ 2. Impairment of antigen processing and delivery^[44]^ 3. Persistent upregulation of PD-L1 expression on the surface of tumor cells^[45]^ 4. Apoptotic resistance in tumor cells^[46,47]^ 5. Induced dormancy and senescence of tumor cells^[48]^ 6. Tumor cells undergo dedifferentiation and EMT^[49]^ 7. MDM2/MDM4 amplification and EGFR mutation^[58]^	Treg cells	1. Competition with conventional T cells for IL-2 via Foxp3^[66,136]^ 2. Secretion of the anti-inflammatory cytokines TGFβ, IL-10, and IL-35^[68,69]^ 3. The dual expression of CD39 and CD73; the CTLA-4-mediated downregulation of CD80 and CD86 on the surface of APCs^[71,73]^ 4. Production of FGL2 to suppress CD8^+^ T cells and APCs through FcγRIIb^[74,137]^ 5. Express PD-1 receptors 6. A spatial ecological niche dedicated to immunosuppression^[76]^
	T cells	1. Release the cytokines IFNγ^[80]^, IL-17^[86,87]^, IL-22^[88,89]^, TNFα^[90,91]^, and IL-6^[92]^ 2. The combination of multiple cytokines, such as TGFβ and TNFα^[80]^ or IFNγ and TNFα^[93]^ 3. The binding of CD27 receptor to CD70 ligand^[94]^
	B cells	IgG4 competes with IgG1 to bind to Fc receptors on the surface of immune effector cells^[107]^
	Fc receptor	The binding of the Fc region of the anti-PD-1 antibody to the macrophage FcγR^[62]^

### Alteration in the tumor cell types following ICI

HPD is a type of primary resistance to immunotherapy, and the mechanism of its occurrence involves alteration in the tumor cell types and the tumor microenvironment. These changes range from enhanced proliferative capacity, invasiveness, and drug resistance of tumor cells to a reduced immunosuppressive capacity in the tumor microenvironment. The tumor cells themselves are altered due to the following reasons: (1) loss of expression of tumor-associated antigens^[43]^; (2) impairment of antigen processing and delivery, including the loss of human leukocyte antigen expression, failing to deliver tumor antigens to the cell surface^[44]^; (3) persistent upregulation of PD-L1 expression on the surface of tumor cells, which competes with ICI for binding to PD-1 receptors on the surface of CD8^+^ T cells and inhibits the anti-tumor immune response^[45]^; (4) apoptotic resistance in tumor cells^[46,47]^; (5) induced dormancy and senescence of tumor cells^[48]^, whereby the tumor cells are temporally controlled and lay the groundwork for future recurrence and metastasis; and (6) tumor cells undergo dedifferentiation and epithelial to mesenchymal transition (EMT)^[49]^.

### MDM2/MDM4 amplification and EGFR mutation

In 2017, Kato *et al*.^[12]^ evaluated 155 patients with advanced tumors and found a 3.9% incidence of HPD. Through Next-Generation Sequencing (NGS), murine double minute 2/4 (MDM2/MDM4) amplification was identified in six patients who had TTF < 2 months and two patients were diagnosed with HPD; in 10 other patients, epidermal growth factor receptor (EGFR) mutations were identified. By multivariate analysis, it was found that MDM2/MDM4 amplification and EGFR mutations were associated with TTF < 2 months. The presence of MDM2 amplification and EGFR mutations in patients with HPD were also found in a clinical study by Singavi *et al*.^[50]^ and Economopoulou *et al*.^[51]^. The MDM2 protein encoded by the *MDM2* gene is a major negative regulator of the p53 protein. MDM2 can ligate to the p53 protein through the E3 ubiquitin ligase, and the ubiquitinated p53 can be transferred to the cytoplasm and targeted for degradation by the proteasome^[52]^. Thus, MDM2 amplification can promote tumorigenesis directly or indirectly through the inhibition of p53. In 2018, Kato *et al*.^[53]^ extended the scope of NGS sequencing to include 102,878 patients with different malignancies and found MDM2 amplification in 3.5% of patients; this was present in a small proportion of patients in most tumor types, and 97.6% of these patients had potentially targetable genomic co-alterations, which suggested that appropriately targeted drugs could be designed to target MDM2 amplification-induced HPD. Fang *et al*.^[54]^ conducted preclinical studies using the MDM2 inhibitor, APG-115. It acts as an indirect p53 activator, suppresses M2 macrophage polarization, and slows tumor invasion and progression, improving anti-tumor immunity to anti-PD-1 treatment. APG-115-mediated p53 activation promoted anti-tumor immunity in TME regardless of the Trp53 status of the tumor itself. Sahin *et al*.^[55]^ also used the MDM2 inhibitor AMG-232 in combination with anti-PD-1 antibody therapy to enhance T cell-mediated killing of tumors regardless of PD-L1 expression. Another MDM2 inhibitor, idasanutlin (RG7388), in combination with cytarabine therapy, is the first to enter phase III clinical trials for AML^[56,57]^.

EGFR is the first identified member of the ErbB family and plays an important role in physiological processes, including cell growth, proliferation, and differentiation. EGFR is also involved in tumor development and immunotherapy-related resistance. A meta-analysis involving 21,047 patients from 35 randomized controlled trials indicated that patients with EGFR wild type had significantly prolonged PFS and OS after treatment with ICI, while those with EGFR mutations did not show any improvement^[58]^. This in part reflected the fact that EGFR mutations are a cause of ICI resistance. The TME in EGFR mutated lung adenocarcinoma was non-inflammatory; interestingly, the non-inflammatory TME had a high infiltration of CD4^+^ Treg cells. EGFR signaling activates cJun/cJun N-terminal kinase and reduces the level of interferon regulatory factor-1; the former increases CCL22 and thereby recruits CD4^+^ Treg cells, while the latter reduces the levels of CXCL10 and CCL5 and, in turn, induces CD8^+^ T cell infiltration^[59]^. In addition, EGFR can upregulate the number of immunosuppressive receptors and induce the secretion of cytokines with immunosuppressive functions [IL-6, IL-10, and transforming growth factor (TGFβ)] from the TME, which in turn leads to ICI treatment resistance^[60]^. To some extent, this may explain the occurrence of HPD in patients with EGFR mutations after ICI treatment; however, the exact mechanism of induction needs to be further elucidated. Other somatic mutations and carcinogenic pathways exist in addition to MDM2 amplification and EGFR mutations. Xiong *et al*.^[61]^ evaluated the mutational and transcriptional characteristics of tumors before and after anti-PD-1 immunotherapy in two patients who acquired HPD. Somatic mutations in recognized cancer genes, including tumor suppressor genes such as *TSC2* and *VHL*, were discovered, as well as transcriptional activation of carcinogenic pathways including IGF-1, ERK/MAPK, PI3K/AKT, and TGFβ.

### Treg cells

Treg cells are important for the maintenance of the body’s immune tolerance. The majority of CD4^+^ Treg cells are produced by the thymus, which accounts for 10% of circulating CD4^+^ T cells. The major transcription factor is Foxp3, which determines the phenotypic and functional characteristics of Treg cells^[62]^. In a normal organism, Treg cells negatively regulate immune cells such as effector T cells to prevent autoimmune overload, while, in tumors, Treg cells exhibit different biological functions^[63]^. Kang *et al*.^[25]^ found significantly higher FoxP3^+^ Treg cells in 74 patients with advanced NSCLC who developed HPD and significantly fewer Treg cells in non-HPD patients (*P *= 0.024). Therefore, PD-1^+^ Treg cells could be an effective biomarker for the identification of HPD^[64]^. Previous studies have shown that high Foxp3^+^ Treg cell infiltration in tumors is significantly associated with poorer OS^[65]^. Foxp3 is a transcriptional repressor of IL-2 that also isolates transcriptional activators acute myeloid leukemia 1 and nuclear factor of activated T-cells outside the nucleus, preventing Treg cells from producing IL-2^[66]^. However, Tregs and conventional CD4^+^ T cells both require IL-2 to survive. As a result, Treg cells compete with conventional T cells for IL-2 via Foxp3 by boosting the expression of CD25 (IL2α), leading to the formation of a high-affinity IL-2 receptor (heterotrimeric complex (IL2Rαβγ)^[67]^.

Treg cells secrete the anti-inflammatory cytokines TGFβ, IL-10, and IL-35 to deplete conventional T cells^[68,69]^. TGF, as a Th1 inhibitor, stimulates the TGFβRI/II receptor on conventional T cells to block IFNγ-induced Th1 activation by inhibiting the expression of two essential Th1 transcription factors, T-bet and IFN regulatory factor 1^[70]^. Indeed, IL-10^+^ and IL-35^+^ Treg cells account for a large proportion of tumors. Gene profiles of conventional T cells exposed to these Treg subtypes were analyzed, and it was discovered that T cells depletion was promoted by IL-35^+^ Treg, but antitumor effects were inhibited by IL-10^+^ Treg^[68]^.

Treg cells, which have the dual expression of CD39 and CD73, block T cell activation by adenosine triphosphate (ATP) and generate adenosine to inhibit T cells. CD39 and CD73, respectively, hydrolyze ATP/ADP to AMP and AMP to adenosine, leading to a large enrichment of adenosine around Treg cells. Adenosine can induce actin cytoskeleton rearrangement and hence function as a chemoattractant for dendritic cells (DCs), causing DCs to congregate towards Treg cells^[71]^. Then, with enhanced leukocyte function-associated antigen-1 stability and expression, Treg cells and DCs create a tight aggregate, decreasing the interaction of T cells to DCs^[72]^. On the other hand, cytotoxic T-lymphocyte-associated protein 4 (CTLA-4) is mediated by Treg cells, resulting in the downregulation of CD80 and CD86 on the surface of antigen-presenting cells (APCs) to restrict the activation of conventional T cells^[73]^. However, it is uncertain whether the capacity of Treg cells to control CD80 and CD86 is simply dependent on CTLA-4 expression.

Treg cells produce fibrinogen-like protein 2 (FGL2) to suppress CD8^+^ T cells and APCs through FcγRIIb. FGL2 is considered a signaling molecule for Treg cells as Foxp3 in Treg stimulates the expression of FGL2^[74]^. The major immunomodulatory effect of FGL2 is mediated via FcγRIIB in APCs. A study has demonstrated that mice lacking the FcγRIIB receptor develop autoimmune glomerulonephritis^[75]^.

Treg cells can also express PD-1 receptors on their surface, and, although blocking the PD-1/PD-L1 axis activates T cells, Treg cells are also highly active, immune function is greatly affected, and anti-tumor immune efficacy is reduced. However, highly activated Treg cells in lymphoid organs resist newly generated anti-tumor T cells, leading to a more attenuated anti-tumor immune effect. These result in uncontrolled tumors, which may lead to HPD. In addition, Murakami *et al*.^[76]^ reported a spatial ecological niche dedicated to immunosuppression which is formed between CD8^+^CD39^+^PD-1^+^ T cells and Foxp3^+^PD-1^+^ Treg cells due to potential interactions between these cells in close proximity following PD-1 blockade in renal cancer. The shift to an immunosuppressive environment is more pronounced in metastatic foci. Anti-CD25 and PD-1 bispecific antibodies are currently used for treatment to deplete Treg cells. Subsequent treatment with anti-PD-1 antibodies may only enhance conventional T cells and CD8^+^ T cells^[77]^. Alternatively, adenosine, a product of Treg cells, could be inhibited by combining the anti-PD-1 antibody with adenosine deaminase for its degradation to inosine, thereby reducing cAMP production to weaken the inhibition of conventional T cells and enhance anti-tumor immunity^[62]^. The possibility of interfering with the systemic immune system is considerably minimized by precisely destroying Treg cells around tumor cells.

### T cells

The function of T cell adaptive immunity is to eliminate tumor cells that positively express antigens^[78]^. However, ICI-enhanced T cell adaptive immune response cannot completely kill tumor cells, as reported in most clinical trials. Even after ICI treatment, adaptive immunity can promote tumor growth directly or indirectly. As a consequence, some researchers believe that enhanced tumor adaptive immune response may be the root cause of HPD in tumor patients after ICI treatment^[42]^. T cell immune response can induce changes in gene expression of tumor cells, such as a downregulation of tumor surface antigens^[79]^ and upregulation of other immune checkpoint ligands^[45]^. However, the underlying mechanism of T cell immune response leading to changes in tumor cells remains changes in tumor cells are still not clear.

The cytokine IFNγ released by T cells may explain a part of the problem. IFNγ, a common cytokine, is involved in several cellular changes, including EMT induction^[80]^. EMT in tumor cells is related to the upregulation of inhibitory checkpoint ligands^[81]^, resistance to cell-mediated cytotoxicity^[82]^, and the production of immunosuppressive effects^[83]^. Furthermore, IFNγ has the ability to upregulate immune checkpoint ligands^[84]^, inducing tumor cell dormancy, apoptosis^[84]^, and hyperplasia.

The same cytokine can play different roles in different environments, depending on the length of time it acts on tumor cells. For instance, prolonged exposure to IFNγ and low levels of the cytokine have been demonstrated to have pro-tumorigenic effects^[85]^. Other cytokines, such as IL-17^[86,87]^, IL-22^[88,89]^, tumor necrosis factor α (TNFα)^[90,91]^, and IL-6^[92]^, are involved in tumor promotion. The combination of multiple cytokines may have a greater tumor-promoting effect than a single cytokine; for example, TGFβ1 leads to demethylation of PD-L1 promoter and TNFα leads to the expression of demethylated promoter and co-induces the overexpression of PD-L1^[80]^. IFNγ and TNFα can co-induce dormancy in tumor cells to promote carcinogenesis^[93]^. However, there is no clear answer as to which T cell subsets are mainly responsible for the release of these cytokines.

In addition to cytokines, some studies report that the binding of CD27 receptor to CD70 ligand can directly promote proliferation and differentiation of tumor stem cells^[94]^ or T cell exosomes to induce EMT and lead to rapid tumor progression^[95]^. Many T cell subsets are involved, including CD4^+^ T cells^[96,97]^, CD8^+^ T cells^[98,99]^, Th1 cells^[100]^, Th2 cells^[101]^, Th17 cells^[102]^, and Th22 cells^[89]^. However, the proportion and spatial distribution of tumor cells and effective infiltration of immune cells may be the watershed response of adaptive immunity when the tumor-immunity balance is broken.

Although there are few studies on non-Treg CD4^+^ T lymphocytes, after ICI treatment, their levels may show an unexpected increase, which can contribute to the occurrence and development of HPD. A prospective study by Arasanz *et al*.^[103]^ included 70 patients with advanced NSCLC who underwent ICI treatment. Early detection of HPD in NSCLC by monitoring T cell dynamics showed a strong expansion of highly differentiated CD28^-^CD4^+^ T lymphocytes (CD4^+^ THD) between the first and second treatment cycles in HPD patients and a significant stratification among HPD patients, non-HDP patients, and effective patients (median 1.525, 1.000, and 0.9700, respectively, *P* = 0.0007). As a consequence, the strong expansion of CD28^-^CD4^+^ T lymphocytes in peripheral blood during the first treatment cycle could provide an early differential feature of HPD induced by ICI in the treatment of NSCLC. These studies suggest that CD8^+^ T cells and Treg cells are involved in the occurrence and development of HPD in TME. However, several innate and adaptive immune cells may be swept into this storm.

### B cells

PD-1 can also be expressed on the surface of B cells. Some studies have pointed out that anti-PD-1 antibodies can increase the activation, proliferation, and production of inflammatory cytokines in B cells^[104]^. However, follow-up studies show that the loss of B cells does not seem to have any effect on the efficacy of ICI treatment^[105]^. The reason for these differences may be due to the existence of different subsets of B cells. The balance among different B cells (resting B cells, activated B cells, Bregs, and other differentiated B cells) determines the ultimate role of B cells in tumor immunity^[106]^. Humoral immunity may play a role in carcinogenesis. Wang *et al*.^[107]^ studied the distribution and mechanism of IgG4 secreted by B cells in the tumor model and found that the increase in B lymphocytes containing IgG4 in cancer tissues and the increase in IgG4 concentration in serum were highly correlated with the poor prognoses of patients with esophageal cancer. Using a mouse model, it was verified that IgG4 competes with IgG1 to bind to Fc receptors on the surface of immune effector cells and suppresses classical immune responses such as antibody-dependent cytotoxicity (ADCC), antibody-dependent phagocytosis, and complement-dependent cytotoxicity. Thus, tumor cell growth was indirectly promoted. Interestingly, nivolumab is essentially IgG4 with a stable S228P mutation and significantly promotes the growth of tumors in mice. However, there are only a few studies on the mechanism of B cells participating in HPD after ICI treatment, and these need further validation.

### Fc receptor

The binding of the Fc region of the anti-PD-1 antibody to the macrophage FcγR consumes M1 macrophages and stimulates their differentiation to M2-like form. This is another clear mechanism of HPD after ICI treatment in addition to Treg cell-mediated inhibition of anti-tumor immunity leading to HPD^[62]^. The antibody consists of F(ab’)_2_ segment bound to the antigen and Fc region bound to FcγR on the surface of immune cells. The binding of the Fc region of IgG antibody to macrophage FcγR triggers the ADCC effect, consumes M1 macrophages and NK cells, and reduces the anti-tumor immune effect^[107,108]^. Other studies have shown that many M2-PD-L1^+^ macrophages were observed in the tumor tissues of NSCLC patients with HPD, which could deplete ICI through Fc-FcγR interaction, induce M2-like differentiation of macrophages, and secrete IL-10 to mediate the HPD occurrence^[109,110]^. The removal of ICI in the Fc region or knockout of FcγR on the surface of macrophages may be a potential research direction for further improvement^[111]^.

## CONCLUSION

HPD occurrence is currently a limitation of ICI treatment and represents the storm-like progression of tumors after ICI administration. The mechanism of HPD is similar to a “tug-of-war” between tumor and anti-tumor effects. Intervention through ICI breaks this balance. It leads to the occurrence of HPD if tumor cells are activated and the anti-tumor effect is inhibited. The side effects of chemotherapy cannot be ignored, although the present incidence of HPD in immune combined chemotherapy has been reduced. One day, we hope to usher in the era of “de-chemotherapy”. Then, it would be necessary to face the problem of HPD due to ICI. Hence, the review provides a significant understanding of the current underlying mechanisms for HPD.

## DECLARATIONS

### Copyright

© The Author(s) 2022.
